# Molecular docking analysis of shatavarins with female hormonal receptors

**DOI:** 10.6026/973206300200775

**Published:** 2024-07-31

**Authors:** Neelima Arora, Amit Kumar Banerjee

**Affiliations:** 1 Department of Biotechnology, Institute of Science, Jawaharlal Nehru Technological University-Hyderabad, Kukatpally, Hyderabad -500085, Telangana State, India; 2 Biology Division, CSIR-Indian Institue of Chemical Technology, Uppal Road, Tarnaka, Hyderabad-500007, Telangana State, India

**Keywords:** Shatavari, shatavarins, shatavarin I, shatavarin IV, hormonal receptor, ayurveda, alternative medicine

## Abstract

Shatavari (*Asparagus racemosus*) has been used for female health problems since ancient times and is useful for treating various female reproductive problems including menopausal problems, hormonal imbalance, lactation, menstrual issues, and others. Shatavarins, the primary phytoconstituents of Shatavari, have high molecular weights and may interact with hormone receptors. We have conducted a molecular docking analysis for different Shatavarins such as Shatavarin I, Shatavarin IV, Shatavarin VI, Shatavarin VII, Shatavarin VIII, Shatavarin IX, and Shatavarin X with different hormonal receptors such as estrogen alpha, beta, and gamma receptor, progesterone receptor, FSH, and LH receptors. The best docking conformations with the highest docking scores, specific interactions and bond formations, and the most important residues of the receptors were identified and reported. The study was successful in providing an initial comparative insight into the binding efficiencies of the Shatavarins for different female hormonal receptors.

## Background:

*Asparagus racemosus*, belonging to the family *Asparagaceae*, is an important Ayurvedic herb found in tropical and subtropical regions of India. It has numerous mentions in several ancient Ayurvedic texts, Siddha, and Unani systems of medicine [1]. *Asparagus racemosus* has been used in treating dysentery, ulcers, nervous disorders, bronchitis, dyspepsia, and weakness [2]. Shatavari, the "Queen of herbs" is known for its use in resolving female reproductive health issues such as painful menstrual bleeding, dysmenorrhea, chronic pelvic pain, infertility, post-menopausal symptoms, vaginal dryness, etc. besides its role in anti-aging and modulation of the immune system. [3]. Shatavari root extract contains several phyto-constituents including Shatavarins, a major group of medicinal compounds that serve as active pharmaceutical ingredients (API) and render medicinal effects [4]. Various Shatavarins, specifically Shatavarin I and Shatavarin IV, extracted from the root of *Asparagus racemosus*, have been isolated and characterized [5-6]. Structurally, Shatavarins are steroidal saponins that are the major bioactive phyto-constituents of Shatavari [7]. Earlier reports have shown that the phyto-constituents of Shatavari have an affinity toward estrogen receptors [8]. Therefore, it is of interest to document the molecular docking analysis of shatavarins derivatives with female hormonal receptors.

## Methodology:

## Receptor and Ligand collection and preparation:

The X-ray crystal structures for the proteins estrogen alpha receptor (PDB ID:3ERD), estrogen beta receptor (PDB ID:1X7J), estrogen gamma receptor (PDB ID:2ZKC), FSH receptor (PDBID:1XWD), LH receptor (PDBID: 7FIJ), progesterone receptor (PDBID: 1A28) were acquired from the RCSB database [9]. These crystal structures were considered as the target receptors after removing the prebound ligand. The selected seven compound structures were taken from the PubChem database [10]. For each receptor protein considered in this study, all seven compounds were considered as ligands for the docking process.

## Lipophilicity and aqueous solubility prediction:

ALOGPS 2.1 tool [11] was used to compute the adsorption, distribution, metabolism, and toxicity of the considered ligand molecules.

## Molecular docking:

The receptor files were used for the respective spheres selection within the receptor during the receptor preparation stage using the DOCK6 program [12]. Hydrogen atoms were eliminated, and UCSF Chimera [13] was utilized to prepare the receptor. The receptor parameters were generated using UCSF Chimera. The AMBER14SB force field was used for parameterization. The DMS module of the DOCK6 package was utilized to calculate the solvent-accessible surface of the ligand binding site with a probe radius of 1.4 å. Receptor spheres were generated through the SPHGEN module of DOCK6, with selection criteria limited to spheres within 10 å from the positions of the prebound ligand coordinates. A grid box enclosing the selected spheres was created, with an additional 5 å added in each dimension. Ligand flexibility was considered in the docking process using the DOCK6 module, and the results were presented as grid scores. Docking was performed for all protein receptors with the seven compounds. Docking scores for each ligand-receptor combination were calculated and tabulated. All visualizations were done using BIOVIA [14].

## Results:

After the initial search for available Shatavarins, the ligand data were collected from the PubChem database. Representatives of each type of Shatavarins were collected for this study.

## Ligand Molecules:

The Shatvarins considered for this study are Shatavarin I, Shatavarin IV, Shatavarin VI, Shatavarin VII Shatavarin VIII, Shatavarin IX, and Shatavarin X (Table 1). Compound ID, molecular formula, molecular weight (g/mol), canonical SMILES, and structures of Shatavarins are presented in Table 1. The molecular weight of selected compounds ranged between 885.0 g/mol and 1067.2 g/mol.

We predicted the lipophilicity of the Shatavarin molecules using the ALOGPS 2.1 program (Table 2). The ALOGPs, ALOGpS, average logS, XLOGP2, and average logP/average logS values were computed based on the SMILES for each compound. The logP value indicates the lipophilicity of drug or drug-like molecules considering water and octane at equilibrium. Therefore, the lop P value is significant for understanding the lipophilicity or lipophobicity of a molecule in a cellular microenvironment. As per Lipinski's rule, the acceptable range of logP is between 0 - 5 [15, 15- 17]. Similarly, logS values represent the aqueous solubility (Table 2). This is important for understanding the solubility of a drug or drug-like compound. The average logS values ranged between -2.95 and -3.48 whereas the XLOGP2 values ranged between 0.20 and 2.75 (Table 2). The observed values were within the acceptable and recommended ranges for lipophilicity and aqueous solubility and agreed with Lipinski's rule of 5 [18].

## Molecular docking:

Docking was done following the methodology mentioned earlier for the respective pockets considered in the receptor proteins. No docking outcome was observed for the estrogen alpha receptor protein; however, the considered ligands were docked for the rest of the target protein molecules (Table 3). The Shatavarin I showed maximum binding affinity (-31.02) towards the estrogen gamma receptor protein followed by estrogen beta and progesterone receptor. The Shatavarin IV ligand molecule also showed a higher affinity (-41.19) towards the estrogen gamma receptor followed by an affinity for the progesterone receptor (Table 3). High affinity for the estrogen gamma receptor was also observed for Shatavari VI (-44.12) and Shatavarin VII (-38.34) (Table 3). However, Shatavarin VIII and Shatavarin IX showed better affinity towards the progesterone receptor, and Shatavarin X showed comparatively higher affinity for the FSH receptor.

**Figure 1:**
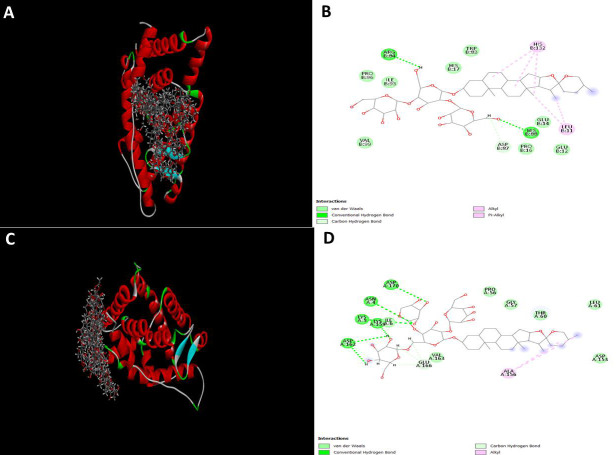
Interactions of (A and B) Estrogen Beta receptor with Shatavarin IX, and (C and D) Estrogen gamma receptor with Shatavarin VIII.

**Figure 2:**
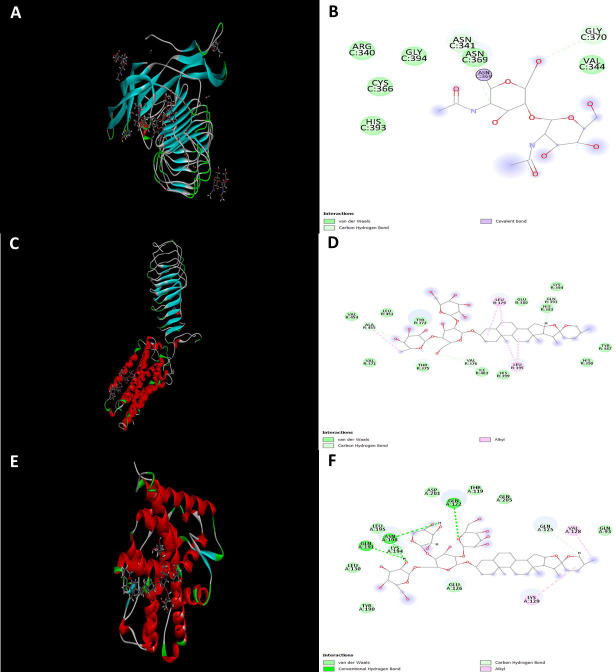
Interactions of (A and B) Follicle-stimulating hormone receptor with Shatavarin VIII, (C and D) Leutinizing Hormone Receptor with Shatavarin VI, and (E and F) Progesterone receptor with Shatavarin VIII.

For each receptor, Shatavarin with the highest binding affinity was analyzed (Figure 1 and Figure 2). It was observed that the estrogen beta receptor showed maximum affinity for Shatavarin IX (-41.15), the estrogen gamma receptor showed high affinity for Shatavarin VIII(-47.65) (Figure 1), the FSH receptor showed the highest affinity for Shatavarin VIII (-48.19), the luteinizing hormone receptor showed the highest affinity for Shatavarin VI(-38.98) (Figure 2), and the progesterone receptor showed the highest affinity for Shatavarin VIII among the considered molecules (Figure 1 and Figure 2).

The specific interactions between the highest docking score containing receptor-ligand pairs were further investigated at the molecular level. The formation of van der Waal bonds, conventional hydrogen bonds, carbon-hydrogen bonds, alkyl, and Pi alkyl bond formations were investigated and presented for the best ligand-protein interactions for each receptor (Figure 1 and Figure 2). The estrogen beta receptor showed bond formation with Shatavarin IX through ArgB:84, ProB:96, IleB:93, ValB:99, HIsB:17, Trpb:83, AspB:87, ProB:16, HisB:88, GluB:14, GluB:12, LeuB:11, and HisB:132. Among these interacting amino acid residues, ArgB:84 and HisB:88 formed hydrogen bonds with the ligand molecule (Shatavarin IX). AspB:87 developed a van der Waal bond with the ligand(FFigure 1B). Similarly, for the estrogen gamma receptor, AspA:170, AsnA:4, IleA:6, LysA:159, LysA:5, and AspA:162 formed hydrogen bonds with the Shatavarin VIII molecule (Figure 1D). Similar results related to the hydrogen bond formation, van der Waal bond formation, and conventional other bond formation for FSH, LH, and progesterone receptors are shown in Figure 2B, Figure 2D, and Figure 2F. The results suggest that Shatavarins have an acceptable range of predicted lipophilicity and aqueous solubility following Lipinski's rule. The comparative binding affinities of different Shatavarins towards the considered target female hormonal receptors suggested the comparative preference of the individual Shatavarin towards specific hormonal receptor protein.

## Discussion:

Natural products are an important source of medicinal compounds globally. Our profound reliance on natural compounds is majorly due to the acceptable safety and efficacy profiles of the natural compounds, abundance in nature, and time-tested applications for various ailments. However, natural compounds should be considered bioactive molecules after strict scientific standardizations and experiments. Time-consuming and cost-intensive experiments often become a barrier during cataloging and exploring medicinal compounds. High-throughput screening help to overcome this barrier [19]. Millions of compounds can be screened in a time-and-cost-effective manner and several additional experimental steps can be skipped. Molecular docking and QSAR analysis, pharmacophore modeling provided substantial evidence for the successful virtual screening of important and potential drug molecules. Shatavari is an important Ayurvedic herb that has several bioactive phytoconstituents such as various Shatavarins which have high molecular weights and large structures [20]. The effects of Shatavari are well-known in improving female health conditions [20]. Shatavari is known to manage and improve female reproductive health, managing symptoms of menopause, anti-oxidant effect, anti-anxiety effect, helps in proper lactation, and other female health-related aspects [3]. Earlier studies reported the possible interactions of Shatavarins with estrogen receptors [8]. Molecular docking analysis showed the comparative efficiency of Shatavarins with available drugs such as Bazedoxifene [8]. In the present study, we have compared the binding efficiency of seven Shatavarins for estrogen, progesterone, FSH, and LH receptors. We have successfully demonstrated the comparative binding affinities of the considered molecules. However, the estrogen alpha receptor considered in this study did not show any binding of the ligands for the existing ligand-bound pocket.

## Conclusions:

A comparative perspective of the receptor binding affinities of different Shatavarins for multiple female hormonal receptors is shown. However, this study is limited to the representative Shatavarins and the hormonal receptor proteins. A detailed virtual screening with all Shatavarins and all hormonal receptor proteins may provide conclusive insight into the comparative interactions of the ligand-receptor complex.

## Figures and Tables

**Table 1:** Details of the compounds considered for the molecular docking in this study.

**Compound Name**	**PubChem ID**	**Molecular Formula**	**Molecular Weight (g/mol)**	**Canonical SMILES**
Shatavarin I	CID_101406647	C_51_H_86_O_23_	1067.2	CC1C2C(CC3C2(CCC4C3CCC5C4(CCC(C5)OC 6C(C(C(C(O6)CO)OC7C(C(C(C(O7)C)O)O)O)O)OC8C(C(C(C(O8)CO)O)O)O)C)C)O C1(CCC(C)COC9C(C(C(C(O9)CO)O)O)O)O
Shatavarin IV	CID_441896	C_45_H_74_O_17_	887.1	CC1CCC2(C(C3C(O2)CC4C3(CCC5C4CC C6C5(CCC(C6)OC7C(C(C(C(O7)CO)OC8C(C(C(C(O8)C)O)O)O)O)OC9C(C(C(C(O9) CO)O)O)O)C)C)C)OC1
Shatavarin VI	CID_101847687	C_45_H_74_O_17_	887.1	CC1CCC2(C(C3C(O2)CC4C3(CCC5C4CCC6C5(CCC(C6)OC7C(C(C(C(O7)CO)OC8C(C(C(C(O8)C)O)O)O)O)OC9C(C(C(C(O9)CO)O)O)O)C)C)C)OC1
Shatavarin VII	CID_101847688	C_45_H_72_O_17_	885	CC1C2C(CC3C2(CCC4C3CCC5C4(CCC(C5)OC6C(C(C(C(O6)CO)OC7C(C(C(C(O7)C)O)O)O)O)OC8C(C(C(C(O8)CO)O)O)O)C)C)OC19CCC(=C)CO9
Shatavarin VIII	CID_101847689	C_50_H_82_O_22_	1035.2	CC1CCC2(C(C3C(O2)CC4C3(CCC5C4CCC6C5(CCC(C6)OC7C(C(C(C(O7)COC8C(C(C(C(O8)CO)O)O)O)OC9C(C(C(CO9)O)O)O)O)OC2C(C(C(C(O2)CO)O)O)O)C)C)C)OC1
Shatavarin IX	CID_101847690	C_45_H_74_O_18_	903.1	CC1CCC2(C(C3C(O2)CC4C3(CCC5C4CCC6C5(CCC(C6)OC7C(C(C(C(O7)CO)OC8C(C(C(C(O8)CO)O)O)O)O)OC9C(C(C(C(O9)CO)O)O)O)C)C)C)OC1
Shatavarin X	CID_101847691	C_47_H_76_O_19_	945.1	CC1CCC2(C(C3C(O2)CC4C3(CCC5C4CCC6C5(CCC(C6)OC7C(C(C(C(O7)CO)OC8C(C(C(C(O8)COC(=O)C)O)O)O)O)OC9C(C(C(C(O9)CO)O)O)O)C)C)C)OC1

**Table 2:** Lipophilicity and aqueous solubility prediction of the compounds

**Compound Name**	**PubChem ID**	**ALOGPs**	**ALOGpS**	**Average logS**	**XLOGP2**
Shatavarin I	CID_101406647	-0.68	-2.95(1.20 g/l)	-2.95	0.2
Shatavarin IV	CID_441896	0.62	-3.48(0.29 g/l)	-3.48	2.75
Shatavarin VI	CID_101847687	0.62	-3.48(0.29 g/l)	-3.48	2.75
Shatavarin VII	CID_101847688	0.09	-3.44(0.32 g/l)	-3.44	2.04
Shatavarin VIII	CID_101847689	-0.45	-2.95(1.16 g/l)	-2.95	0.76
Shatavarin IX	CID_101847690	0.05	-3.18(0.59 g/l)	-3.18	1.84
Shatavarin X	CID_101847691	0.39	-3.45(0.34 g/l)	-3.45	2.36

**Table 3:** Obtained docking scores observed between the ligands and the receptors.

**Ligands**	**Estrogen Alpha receptor (PDBID:3ERD**)	**Estrogen Beta receptor (PDBID: 1X7J)**	**Estrogen Gamma receptor (PDBID: 2ZKC)**	**FSH receptor (PDBID: 1XWD)**	**LH receptor (PDBID: 7FIJ)**	**Progesterone receptor (PDBID: 1A28)**
Shatavarin IV (CID_441896)	Not Docked	-22.42	-41.19	-33.23	-24.04	-36.52
Shatavarin I (CID_101406647)	Not Docked	-29.6	-31.02	-26.66	-12.99	-28.62
Shatavarin VI (CID_101847687)	Not Docked	-38.53	-44.12	-43.85	-38.98	-38.47
Shatavarin VII (CID_101847688)	Not Docked	-28.8	-38.34	-37.02	-13.17	-35.75
Shatavarin VIII (CID_101847689)	Not Docked	-27.46	-47.65	-48.19	Not Docked	-48.7
Shatavarin IX (CID_101847690)	Not Docked	-41.15	-41.08	-39.07	-36.35	-43.19
Shatavarin X (CID_101847691)	Not Docked	Not Docked	-33.97	-39.85	-36.67	-37.2
